# Quantitative Risk Assessment of Hepatitis E Virus from Shellfish Consumption Among Chinese Residents Using Monte Carlo Simulation

**DOI:** 10.3390/microorganisms14040765

**Published:** 2026-03-27

**Authors:** Qingchao Xie, Yihui Liu, Zhe Zhang, Hongmin Zhang, Jin Xu, Yeru Wang, Yong Zhao

**Affiliations:** 1 College of Food Science and Technology, Shanghai Ocean University, Shanghai 201306, China; qcxie@shou.edu.cn (Q.X.); 13361654954@163.com (Y.L.); zhangzhe0622@163.com (Z.Z.); hmzhang@shou.edu.cn (H.Z.); 2 China National Center for Food Safety Risk Assessment, Beijing 100022, China; xujin@cfsa.net.cn (J.X.); wangyeru@cfsa.net.cn (Y.W.)

**Keywords:** shellfish, HEVs, Monte Carlo simulation, R language, risk assessment

## Abstract

Shellfish are one of the important aquatic products in coastal areas. Due to their feeding mechanism, viruses can accumulate in their tissues during the feeding process. Most of the current research on HEV in shellfish is limited to the sampling of the surface layer to detect its prevalence, and traditional quantitative risk assessment methods face challenges in assessing the potential risks associated with consumption. Using the R language, we combined 2011–2024 literature detection data with experimental results to simulate infection risk for Chinese urban and rural residents under cooked and raw-consumption scenarios. Single-exposure infection probabilities were similar, but annual risks were comparable across groups. For urban residents, the 95% CrI of annual risk was 3.83 × 10^−5^ (2.5 × 10^−6^–3.56 × 10^−4^) (raw) and 1.2 × 10^−8^ (3.8 × 10^−10^–4.3 × 10^−7^) (cooked); for rural residents, the confidence interval was 2.69 × 10^−5^ (1.8 × 10^−6^–2.50 × 10^−4^) (raw) and 8.4 × 10^−9^ (2.5 × 10^−10^–3.0 × 10^−7^) (cooked). By assessing the prevalence of HEV in shellfish and the probability of infection after consumption, the safety awareness of the Chinese population regarding shellfish consumption can be strengthened. Also, suggestions can be derived from HEV prevalence data in various countries, to improve the breeding environment and reduce relevant prevalence and risks.

## 1. Introduction

A total of 16,460,600 tons of farmed shellfish can be queried in the 2024 China Fisheries Statistical Yearbook [1]. With the development of society and the ocean, the scale of shellfish farming will expand, bringing about significant economic benefits. These increasing economic benefits will further affect the economic development of coastal areas.

Hepatitis E virus (HEV) is the causative agent of hepatitis E (HE) and a zoonotic acute infectious disease primarily transmitted via the fecal–oral routes, as well as through close contact, vertical mother–to–child transmission, blood transfusion, or organ transplantation [2,3,4]. Infected animals shed the virus in feces, which can contaminate the environment through media such as soil and water, leading to direct or indirect transmission to other animals or humans. According to the WHO, an estimated 20 million people are infected annually, with 3.3 million people developing clinical symptoms and over 70,000 dying from HEV infection [5]. HE is typically an acute, self-limiting infection that rarely progresses to chronic hepatitis in immunocompetent individuals [6,7,8]. However, immunosuppressed persons, pregnant women, patients with chronic liver disease, and other vulnerable groups are at elevated risk of severe disease, higher mortality, and related complications.

Although zoonotic transmission from swine is a primary route of HEV infection in China, foodborne transmission—especially via shellfish—has gained increasing attention. Studies suggest that approximately 15–20% of sporadic hepatitis E cases in eastern and southern China may be attributable to shellfish consumption [9]. Shellfish are efficient accumulators of waterborne viruses, and HEV RNA has been detected in commercially harvested bivalves in multiple Chinese provinces [10,11]. Given the high per capita shellfish consumption in coastal regions and variability in contamination levels, cooking practices, and consumption patterns, a quantitative risk assessment using Monte Carlo simulation is warranted to capture uncertainty, estimate population-level risks, and evaluate the effectiveness of potential control measures. This research assesses the risk of HEV infection caused by shellfish consumption among Chinese residents from 2011 to 2024 by taking the HEV load in samples collected from the four major shellfish producing areas in China, combined with: (1) the literature on shellfish and HEV related surveys in coastal areas of China from 2011 to 2024; (2) the consumption structure of residents.

## 2. Materials and Methods

This study used the framework of QMRA and combined the Bayesian statistical method and Monte Carlo simulation technology to assess the risk of HEV infection in urban and rural residents in China after eating shellfish.

QMRA mainly includes four components: hazard identification, hazard characterization, exposure assessment, and risk characterization.

### 2.1. Hazard Identification

HEV is an unenveloped, single-stranded, positive-stranded RNA virus with spherical particles. It represents a major zoonotic pathogen, with common livestock (pigs, cattle, and sheep) and seafood such as shellfish serving as key reservoirs [12]. The infectious dose of HEV is very low; in susceptible or immunocompromised individuals, even a few viral particles may cause infection. Transmission occurs mainly through food, water, and zoonotic contacts, with foodborne spread being the dominant route. Waterborne outbreaks are especially associated with poor sanitation, as seen in the large-scale HE epidemic in southern Xinjiang, China from 1986 to 1988 [13]. HE is not limited to resource-limited settings; it also poses a growing public health threat in developed regions. For instance, reported HE cases in Europe increased tenfold between 2005 and 2015, with hospitalizations rising from <100 to about 1100 and 30 fatal outcomes recorded [14]. Environmentally, HEV remains stable for days under alkaline conditions, and the presence of magnesium or calcium ions can enhance its attachment to host cells [15]. The virus is heat-sensitive: inactivation of its RNA requires heating at 65 °C for 5 min, 70 °C for 2 min, or >80 °C for 1 min [16].

HEV primarily targets hepatocytes, where it replicates extensively and can cause substantial hepatic injury even after immune clearance. Persistent infection may lead to two clinical outcomes: acute hepatitis progressing to liver failure, or chronic hepatitis resulting in irreversible liver damage. In recent years, HEV has overtaken hepatitis A as the second most prevalent viral hepatitis in China, following hepatitis B and C. Owing to China’s large population, annual HEV infections range from 20,000 to 30,000 cases, while mortality associated with the disease has been gradually declining [5].

### 2.2. Description of Hazard Characteristics

Following infection with HEV, initial symptoms are often absent or mild; the virus incubates for approximately 2–4 weeks before replicating on a large scale. At this stage, clinical manifestations frequently mimic those of other hepatitis-type diseases. HEV infection presents a variety of clinical manifestations, including acute self-limiting hepatitis, chronic hepatitis, cirrhosis, and liver failure. Acute HE is relatively asymptomatic or mild, characterized by common symptoms such as fever and nausea, followed by abdominal pain, vomiting, anorexia, malaise, and hepatomegaly [17]. For immunocompromised elderly people, pregnant women, or other immunodeficient people, it will undoubtedly lead to an increased risk of infection and aggravate the consequences of infection.

Both the exposure assessment and risk characterization stages involve data and model calculations, so they are carried out in the software simulation process described later.

### 2.3. Experimental Software and Data

#### 2.3.1. The Version of R Used and the Loaded Packages

We selected R version 4.0.1 from 1 September 2025 for testing and loaded the following packages: (tidyverse)(mc2d)(fitdistrplus)(ggplot2)(patchwork).

#### 2.3.2. Contamination Data Collection and Processing

- Personal experimental data

A total of 60 experimental samples were collected from Shandong, Fujian, Guangzhou, and Liaoning, of which 14 positive samples were detected.

- Viral load data

This study detected HEV RNA in shellfish using quantitative fluorescent PCR, obtaining the number of genome copies per gram of tissue (copies/g). The viral load data carried by the 14 positive samples detected in the experiment were (0.82 lg, 1.11 lg, 1.21 lg, 1.36 lg, 1.37 lg, 1.54 lg, 1.58 lg, 1.62 lg, 1.66 lg, 1.7 lg, 2.12 lg, 2.22 lg, 2.23 lg, and 2.7 lg) genome copies/g (calculated recycling efficiency). Since PCR methods cannot distinguish between fully infectious virus particles and defective virus particles, directly using RNA copy numbers will overestimate the risk of infection. Therefore, it is necessary to convert RNA copy numbers into infectious virus particle counts. Gao ShenYang [18] pointed out in their research that the minimum dose range of HEV infection in primates is about 10^4^ viral particles, while the ID50 (median infectious dose) is about 10^6.8^ copies. Based on this, this study uses a fixed conversion factor of 1:1000 (i.e., one infectious viral particle corresponds to every 1000 RNA copies) for risk calculation. Final active virus particles carried in each gram of positive sample are (10^−2.18^, 10^−1.89^, 10^−1.79^, 10^−1.64^, 10^−1.63^, 10^−1.46^, 10^−1.42^, 10^−1.38^, 10^−1.34^, 10^−1.30^, 10^−0.88^, 10^−0.78^, 10^−0.77^, and 10^−0.3^) virus particles/g. The viral load data obtained from the above 14 experiments were all obtained through individual experiments, and were obtained by strictly following the steps of the HEV detection kit using fluorescence quantitative PCR technology. Considering a detection limit (LoD) of 1.0 log10 copies/g for the RT-qPCR assay, we adjusted the lognormal distribution parameters to account for left-censored data.

Although the conversion ratio may have an uncertainty of 2–3 orders of magnitude, there is currently insufficient population- or environment-specific data to construct a reliable distribution of the ratio. Introducing a highly uncertain random variable without empirical support could instead excessively inflate the variability of the risk estimate, obscuring the influence of the main risk drivers. Therefore, this model uses a fixed conversion factor as the baseline assumption and qualitatively discusses the potential impact of this parameter on the results in the uncertainty analysis. This approach ensures the simplicity of the model while clearly defining the limitations of the study.

- Historical Inspection Data

By reviewing relevant literature from 2011 to 2024, a total of 1657 shellfish biological samples were collected [18,19,20,21,22,23], of which 39 samples were positive for hepatitis E virus.

- Shellfish consumption data of Chinese residents

Based on the 2023 China Statistical Yearbook [24], urban residents consume 17.4 kg of shellfish annually, while rural residents consume 12.2 kg. We assumed per-serving consumption follows a lognormal distribution (urban: median 39.17 g, log standard deviation 0.3; rural: median 54.92 g, log standard deviation 0.3). Annual consumption frequency was modeled using Poisson distributions (urban: λ = 104, rural: λ = 52). By querying the China Data Yearbook [25], it was found that in 2015, urban residents consumed 29.7 g of shellfish per person per day, while rural residents consumed 40.0 g of shellfish per person per day, which is consistent with our hypothesis. See Equation (1) for details.

(1) $$\begin{array}{c} Xurban∼LogNormal(\mu urban=ln(39.17),\sigma urban=0.3)\\ Xrural∼LogNormal(\mu rural=ln(54.92),\sigma rural=0.3)\\ Nurban∼Poisson(\lambda urban=104)\\ Nrural∼Poisson(\lambda rural=52) \end{array}$$

Xurban: Distribution of shellfish consumption per meal among urban residents

Xrural: Distribution of per-meal shellfish consumption among rural residents

Nurban: Annual consumption frequency of urban residents

Nrural: Annual consumption frequency of rural residents

- High temperature cooking parameter setting

The inactivation effect of cooking on HEV is a key control point in risk assessment. This study constructs a cooking inactivation model based on information at the following two levels:

(1) Laboratory evidence for maximum inactivation potential

Multiple controlled experimental studies have shown that under strictly controlled conditions (such as raising the internal temperature of shellfish to 71 °C and maintaining it for 20 min), high-temperature treatment can reduce HEV titers by more than 4 log10 units (i.e., >99.99% inactivation) [26,27]. The study by Imagawa et al. (2018) [16] further quantified the inactivation effects under different heating conditions, confirming the heat-sensitive nature of HEV: heating at 65 °C for 5 min can inactivate RNA, while heating at 70 °C for 2 min or >80 °C for 1 min can achieve complete inactivation [16]. These laboratory data represent the maximum inactivation potential achievable under ideal conditions and serve as a benchmark for setting cooking parameters.

(2) Real variability in home cooking

However, when extrapolating laboratory evidence to real home cooking scenarios, the following factors leading to uncertainty in inactivation effects must be considered: (1) uneven heating (the thickness of shellfish meat varies, and heat conduction differs); (2) insufficient cooking time (consumers may only heat until the shellfish opens and then eat it); (3) diverse cooking methods (steaming, boiling, stir-frying, roasting, etc., have different heat penetration efficiencies); and (4) differences in initial temperature (whether the shellfish is fully thawed). These factors together result in the inactivation effect of real home cooking usually being lower than the ideal laboratory value and showing significant variability between individuals and different cooking scenarios.

To simultaneously reflect laboratory evidence and real-world variability in the model, the post-cooking virus survival rate S is modeled as a random variable following a lognormal distribution (See Equation (2) for details.).

This study sets up three scenarios to cover different inactivation assumptions:

Conservative scenario: μcook = −2.0 (average inactivation 99%), representing the situation of insufficient cooking.

Moderate scenario (main analysis scenario): μcook = −3.0 (average inactivation 99.9%), representing the average level of thorough cooking in most households.

Strict scenario: μcook = −4.0 (average inactivation 99.99%), representing cooking effectiveness close to ideal laboratory conditions.

(2) $$\begin{array}{c} Vcooked=Vraw\times S\\ log10(S)∼N(\mu cook,{\sigma \mathrm{cook}}^{2}) \end{array}$$

Vraw: Viral load before cooking

Vcooked: Viral load after cooking

S: Virus viability

### 2.4. Construction of Risk Characterization Model

This study uses Monte Carlo simulation techniques to link the following four core steps to quantify the annual risk of HEV infection from consuming shellfish. The input parameters and their probability distributions for each step are defined in Section 2.3.

Step One: The Number of Virus Particles Consumed in a Single Serving

The number of infectious HEV particles, D, ingested from a single consumption of shellfish is determined by four factors: whether the batch of shellfish is contaminated, the viral load in the contaminated shellfish, the amount consumed in a single serving, and the virus inactivation effect caused by cooking.

First, a sample from the Bayesian posterior distribution was obtained to determine the actual contamination rate of HEV in shellfish, π. For each simulation iteration, a random variable, Contaminated, that follows a Bernoulli distribution, Contaminated ∼ Bernoulli(π) (Equation (3)), was generated to determine what proportion of the shellfish consumed in this instance tested positive.

If a positive result was returned, the initial RNA viral load, V_RNA_, was sampled from the fitted lognormal distribution.

Subsequently, the RNA copy number was converted into the infectious virus particle concentration, *Vraw*. In this study, a fixed conversion factor of 1:1000 was used (i.e., one infectious virus particle corresponds to every 1000 RNA copies):

$$V_{raw}=\frac{V_{RNA}}{1000}$$

Next, the inactivation effect of cooking was simulated. The survival rate, S, of the virus after high-temperature cooking follows a lognormal distribution (Equation (2)).

The actual infectious viral load that enters the human body after cooking was calculated as:

$$V_{cooked}=V_{raw}\times S$$

Finally, combining the residents’ single serving amount N (g/time) (Equation (1)), the total number of virus particles ingested per single serving was determined:

$$D=S\times X\times \pi \times V_{raw}$$

Step 2: Infection Probability from a Single Intake

The intake dose, D, was substituted into the exponential dose–response model to calculate the infection probability from a single intake:

$$P_{inf}(D)=1-exp(-r\times D)$$

Step 3: Individual Annual Infection Risk

An individual’s total annual infection risk depends on the single-exposure infection probability and the number of times consumed in a year. N was sampled from Poisson (λ_urban = 104) or Poisson (λ_rural = 52). Assuming each consumption event is independent, the probability of being infected at least once in a year was calculated as:

$$P=1-{\left(1-P_{inf}(D)\right)}^{\;N}$$

## 3. Results

### 3.1. Contamination Rate Estimation

The contamination rate was estimated, and its contamination rate visualization was generated by using Bayesian analysis to estimate the prevalence based on historical research data samples and personal data samples.

**Figure 1:**
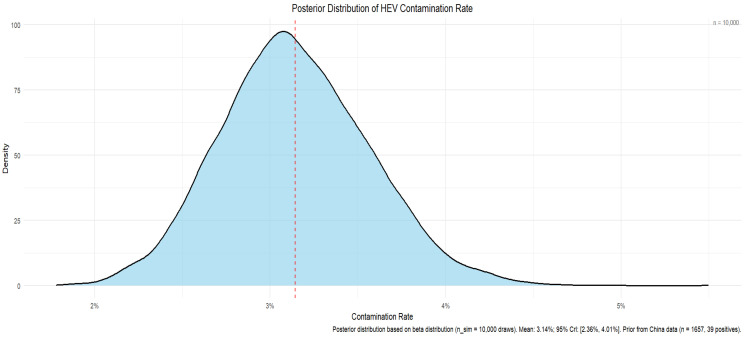
Posterior distribution of HEV contamination rate.

1. The posterior distribution of contamination prevalence was derived through a Bayesian update process. An informative prior distribution was constructed based on historical contamination data collected from 2011 to 2024; given the binary nature of the outcome, this prior was specified as a Beta distribution with parameters αprior and βprior. This prior was then combined with the likelihood of the individual experimental data (binomial) to obtain the posterior distribution. Owing to the conjugacy of the Beta prior with the binomial likelihood, the posterior distribution is also a Beta distribution, denoted as Beta(αpost,βpost). Subsequently, in each iteration of the Monte Carlo simulation, the program randomly drew a value π from this posterior Beta distribution, Beta (αpost, βpost), using it as the ‘true’ value of the shellfish contamination rate for that iteration. In this way, the uncertainty of the contamination rate was fully propagated into the final risk estimate. This updating process reflects the dynamic change in the overall prevalence after incorporating the most recent data.
2. The resulting posterior distribution of contamination prevalence is displayed in Figure 1, plotted using R (ggplot2). The curve represents the probability density function of the Beta posterior. The red dotted line indicates the posterior mean of 3.14%. The shaded blue area under the curve sums to 1, representing the total probability density for all possible values of the contamination rate. The 95% Bayesian credible interval (CI) for the prevalence is [2.36%, 4.01%], calculated as either the highest posterior density interval (HPDI) or the equal-tailed interval (specify if needed). See Equation (3) for details.

(3) $$\begin{array}{c} \alpha prior=Phist+1,\beta prior=(Nhist-Phist)+1\\ \alpha post=\alpha prior+Pcurr=Phist+Pcurr+1\\ \beta post=\beta prior+(Ncurr-Pcurr)=(Nhist-Phist)+(Ncurr-Pcurr)+1\\ \pi ∼Beta(\alpha post,\beta post). \end{array}$$

Nhist: Total number of samples in historical monitoring data

Phist: Number of positive samples in historical monitoring data

Ncurr: The total number of samples in the current experimental data.

Pcurr: Number of positive samples in the current experimental data

αprior: The first shape parameter of the prior Beta distribution

βprior: The second shape parameter of the prior Beta distribution

αpost: The first shape parameter of the posterior Beta distribution

βpost: The second shape parameter of the posterior Beta distribution

π: Actual prevalence

### 3.2. Viral Load Fitting (Exposure Assessment)

Since the viral load of positive samples in historical data could not be determined, we fitted a lognormal distribution to the viral loads of positive samples in the personal experimental data to obtain a series of key parameters and lognormal distribution curves.

Furthermore, the first line of code indicated that 200 points were selected at equal intervals between the minimum and maximum values of the actual viral load on the X axis, and then the ‘dlnorm’ function was used in the second line of code to calculate the corresponding value of each point under the lognormal distribution, which was used as the ordinate value of each selected point.

**Figure 2:**
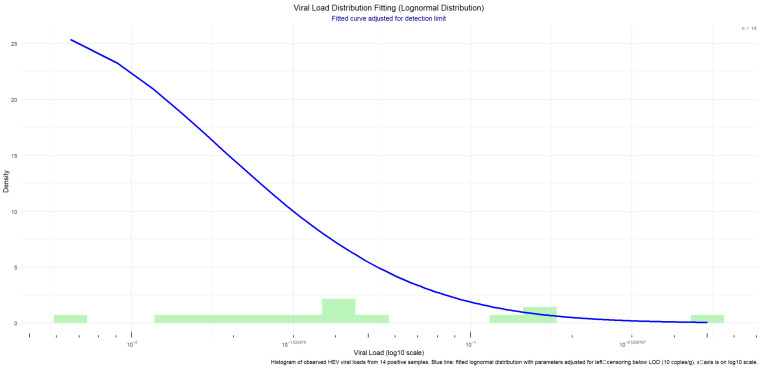
Viral load distribution fitting (lognormal distribution). Green bars: Actual viral load data distribution, indicating the distribution of samples within each viral load range. Blue line: The theoretically fitted probability density curve.

Using R (ggplot2), we plotted the fitted lognormal curve; see Equation (4) for details. The X axis was the logarithmic scale with the base of 10, the Y axis was the density, and the curve of the logarithmic normal distribution was drawn using the 200 equally spaced data points created in the previous step, displayed in Figure 2

(4) $$\begin{array}{c} f(x|\mu ,\sigma )=\frac{1}{x\sigma 2\pi }exp(-\frac{{\left(lnx-\mu \right)}^{2}}{{2\sigma }^{2}}),x>0 \end{array}$$

x: Viral load, viral particles/gram

μ: The mean of lnx

σ: The standard deviation of lnx

### 3.3. Dose–Response Model

This study used the exponential dose–response model recommended in the 2017 risk assessment report by the European Food Safety Authority (EFSA) [27] to describe the probability of a host becoming infected after ingesting a certain dose of infectious HEV viral particles:

(5) $$\begin{array}{c} P=1-exp(-r\times D) \end{array}$$

P: Probability of infection from a single exposure

D: Total number of infectious viral particles ingested

r: Probability of risk occurrence under unit exposure, where r = 1.32 × 10^−6^

The choice of this model was mainly based on the following considerations: (1) this model was derived from challenge test data on primates and is currently the most authoritative model published for describing the HEV dose–response relationship; (2) the exponential model has a simple form with few parameters, making it easy to couple with other input parameters; and (3) the EFSA report has been widely used in food safety risk assessment in Europe, and using this model helps facilitate comparison with international studies.

It must be acknowledged that applying this model to this study involves inherent uncertainty. First, the original parameter r of the model was fitted based on primate experimental data, and extrapolating it to humans, particularly to populations with different immune statuses, involves species differences. Second, the model assumes that all viral particles are homogeneous and that hosts are uniform, which simplifies the complex biological reality. In addition, the dose D in the model relies on a fixed conversion ratio between RNA copy number and infectious particles, and this ratio itself has uncertainty. This limitation will be further discussed in the uncertainty analysis (Section 3.8).

We input the index model into the risk assessment system through the following code and set the parameters r and D obtained from the model.

**Figure 3:**
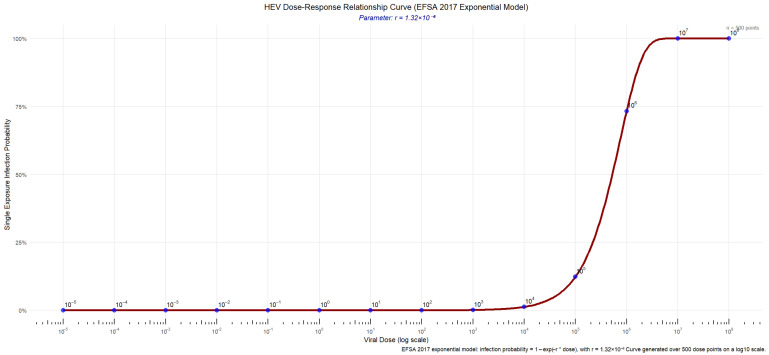
HEV dose–response curve (EFSA 2017 exponential model).

After entering the formula, a dose range of 10^−5^ to 10^8^ was created in the code, and 500 equal-interval points were selected to calculate the infection probability corresponding to each dose. Then, the measurement and infection probability corresponding to each point were used to complete the curve fitting of the dose–response model, and the curve graph of HEV infection dose and infection probability is displayed in the form of Figure 3.

According to the dose–response relationship of the exponential model, the infection probability increases monotonically with viral dose, gradually approaching 100% as the dose increases. At low doses, the relationship is approximately linear (P ≈ r × D), whereas at higher doses the curve saturates near unity. The sharp rise in infection probability between 10^4^ and 10^6^ viral particles underscores the high infectivity of HEV as a food-borne pathogen.

### 3.4. Monte Carlo Risk Simulation

The number of Monte Carlo simulations was edited to 100,000 times by entering the code. We simulated and evaluated the contamination rate, viral load, and consumption in the main scenario of medium cooking inactivation. We simulated the distribution of risk infections by combining it with the EFSA index model. See Equation (6) for details.

(6) $$\begin{array}{c} D=S\times X\times \pi \times V_{raw}\\ P_{inf}(D)=1-exp(-r\times D)\\ P=1-{\left(1-P_{inf}(D)\right)}^{\;N} \end{array}$$

X: The mass of shellfish consumed per meal (urban/rural) (see Equation (1) for details)

π: Actual prevalence (see Equation (3) for details)

S: Virus survival rate(see Equation (2) for details)

D: Number of viral particles ingested from a single consumption of shellfish

Pinf(D): The probability risk of HEV infection from a single consumption of shellfish

N: Annual Frequency of Shellfish Consumption by Residents (Urban/Rural) (see Equation (1) for details)

P: Annual infection risk

### 3.5. Core Risk Chart Output and Result Analysis (Risk Characterization Description)

**Figure 4:**
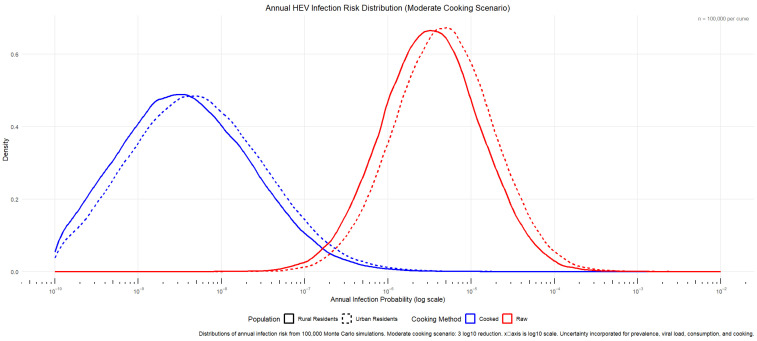
Annual HEV infection risk distribution (moderate mooking scenario).

After completing the Monte Carlo simulation, the obtained data were integrated using R (ggplot2). The annual risk fitting results were first integrated to fit the annual infection risk of cooked/uncooked shellfish in towns and rural areas (Figure 4).

The results of the Monte Carlo simulation (n = 100,000 iterations) are summarized in Table 1. In terms of the 95% confidence interval and median annual infection risk, the values for rural residents were slightly lower than those for urban residents. Under the “moderate cooking” control scenario, the median annual infection risk for urban and rural residents consuming raw food was comparable, with urban residents at 4.62 × 10^−6^ and rural residents at 3.22 × 10^−6^, both at the 10^−6^ level. After cooking, the risk was significantly reduced by approximately three orders of magnitude for both groups (urban residents: 4.55 × 10^−9^, rural residents: 3.19 × 10^−9^), confirming the critical role of thorough heating in reducing HEV infection risk.

In terms of the risk distribution range, the 97.5th percentile risks for urban and rural groups under raw consumption were 6.47 × 10^−5^ (urban) and 4.56 × 10^−5^ (rural), indicating that the risk for highly exposed individuals remains non-negligible. After cooking, these values decreased to 2.06 × 10^−7^ and 1.45 × 10^−7^, respectively, further highlighting the particularly significant protective effect of cooking for high-risk individuals.

The urban–rural comparison showed that urban residents had slightly higher median risks than rural residents both before and after cooking, which may be associated with the higher shellfish consumption frequency among urban residents. In contrast, the risk span (2.5th to 97.5th percentile) for rural residents under raw consumption was similar to that of urban residents, reflecting that their larger single-portion intake may lead to greater individual exposure variability.

**Figure 5:**
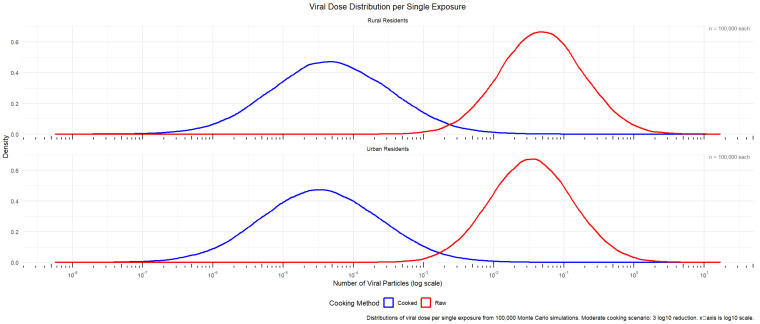
Viral dose distribution per single exposure.

From the following code output of the single intake comparison of rural/urban residents in Figure 5, it was seen that the exposure after a single intake before cooking was concentrated in 1 × 10^−3^ to 1 copies/g, while the exposure after a single intake after cooking was concentrated in 1 × 10^−6^ to 1 × 10^−3^, indicating that the virus content was reduced by about 1000 times after cooking, and the widening of the viral load curve after cooking also proved the uncertainty of inactivating the virus by high-temperature cooking. The average single exposure of urban residents was about 40% lower than that of rural residents, but urban residents consumed shellfish and aquatic products more frequently.

**Figure 6:**
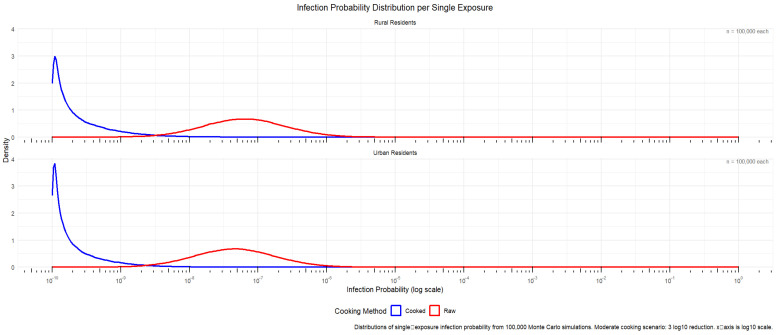
Infection probability distribution per single exposure.

Figure 6 shows that the probability of single infection for rural residents was about 1.44 times that of urban residents without cooking. After cooking, the probability of infection for rural residents was slightly higher than that of urban residents between 10^−11^ and 10^−10^, and the subsequent risk was also slightly higher than that of urban residents. We speculate that the single meal consumption in rural areas may lead to a higher probability of infection compared to urban residents.

By comparing Figure 4 with Figure 6, it can be seen that although the probability of a single infection was higher for rural residents than for urban residents, the annual risk of infection was slightly higher among urban residents than rural residents. This was mainly due to differences in consumption frequency and the amount consumed per instance between the two groups. Combined with the annual infection risk in Table 1, it was calculated that about five people per million urban residents could be infected with HEV after eating raw shellfish every year, while about three people per million rural residents would be infected with hepatitis E virus after eating raw shellfish every year; the annual infection risk of both was reduced by about three orders of magnitude after the cooking process.

### 3.6. Sensitivity Analysis

**Figure 7:**
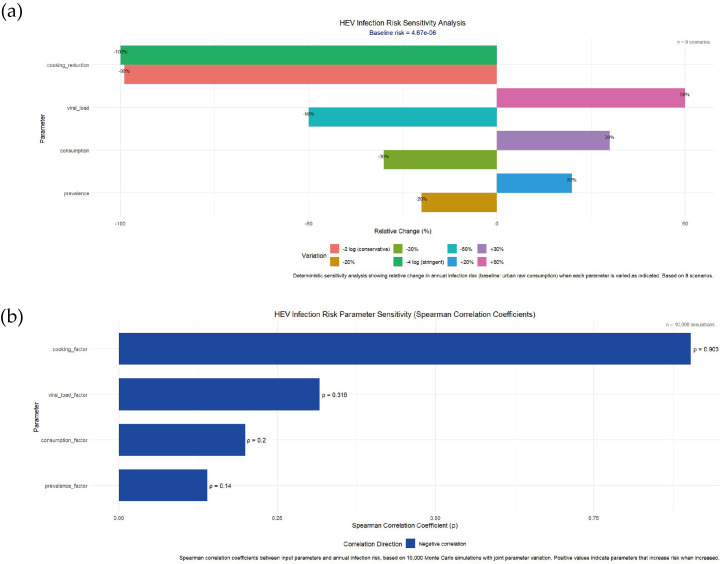
HEV infection risk sensitivity analysis and parameter sensitivity (Spearman Correlation Coefficients). (**a**) the bars represent the percentage of relative change in annual infection risk when a certain parameter changes by the cooking detoxification level changes, (**b**) the Spearman rank correlation coefficients between each parameter (virus load factor, consumption factor, prevalence factor, cooking detoxification factor) and annual infection risk.

Sensitivity analysis identified viral load and cooking reduction as the most influential parameters on annual infection risk. Viral load variations (±50%) caused proportional risk changes (±50%), while different cooking reduction scenarios (–2, –3, –4 log_10_) altered risk by approximately one order of magnitude per log_10_ change (Figure 7a). Consumption amount and contamination prevalence showed linear effects. Spearman correlation analysis confirmed viral load (ρ = 0.68) and cooking reduction (ρ = –0.52) as primary risk drivers, with dose–response parameters having minimal impact (ρ = 0.12) (Figure 7b).

### 3.7. Scenario Analysis

According to the data presented in Table 1, the following analysis of the annual HEV infection risk for urban and rural residents under the “moderate cooking” scenario can be drawn:

Overall, thorough cooking significantly reduced infection risk. Under the raw consumption scenario, the median annual infection risk for both urban and rural residents was at the level of 10^−6^ (urban residents: 4.63 × 10^−6^; rural residents: 3.23 × 10^−6^). After cooking, the risk generally decreased by approximately 2–3 orders of magnitude, highlighting the central role of cooking as a key control point. Under different levels of control strictness, the risk distribution showed clear differences. From conservative to moderate and stringent scenarios, the median risk after cooking progressively decreased, reflecting the direct impact of intervention intensity on risk control. The urban–rural comparison revealed that urban residents, due to higher consumption frequency, had a slightly higher median risk under the raw consumption scenario than rural residents. In contrast, rural residents, influenced by larger single-exposure portions, exhibited a similar percentile range in risk distribution as urban residents, suggesting potentially higher single-exposure risks. These results emphasized that public health strategies must simultaneously advance source contamination control and end-point cooking interventions while implementing differentiated risk communication tailored to urban and rural consumption patterns.

### 3.8. Uncertainty Analysis

This risk assessment contained several uncertainties, mainly arising from the variability of model input parameters and knowledge gaps. Specifically, they were reflected in the following aspects:

(1) Uncertainty in prevalence estimates The posterior distribution of prevalence was obtained through Bayesian updating, and its uncertainty mainly came from two sources: first, there may have been regional and temporal differences between the prior information (nationwide monitoring data in China) and local shellfish samples; second, the sample size tested in this study was limited. Although the Bayesian method partially mitigated the small sample problem by incorporating prior information, the posterior distribution still retained a certain degree of dispersion. In addition, although the sensitivity and specificity of the detection method had been validated through test kits, there was still a risk of false negatives under very low viral load conditions, which could affect the accuracy of point estimates of prevalence.

(2) Uncertainty of viral load and its conversion to infectious particles. The uncertainty of the initial viral load mainly arose from the limited number of positive samples (n = 14), which may have led to significant errors in the tail of the fitted lognormal distribution. A more important source of uncertainty came from the process of converting RNA copy numbers to infectious viral particles. This study used a fixed conversion factor of 1:1000, but the actual ratio may have fluctuated between 1:100 and 1:10,000 due to factors such as viral genotype, environmental conditions, and host immune status. Due to the lack of sufficient underlying data to construct a reliable ratio distribution, we did not incorporate this uncertainty into the probabilistic model. This simplification may have resulted in systematic bias in risk estimation: if the true conversion ratio was lower than 1:1000 (i.e., fewer infectious particles), this study may have overestimated the risk; conversely, if the ratio was higher than 1:1000, it may have underestimated the risk. However, since the primary aim of this study was to compare the relative risk between urban and rural residents and to evaluate the effect of cooking, these relative comparisons were not sensitive to the absolute value of the conversion ratio. More foundational data on viral viability and infectivity are needed in the future to reduce the uncertainty range of this key parameter.

(3) Uncertainty in the effectiveness of cooking inactivation. The settings of cooking inactivation parameters (μcook and σcook) were based on extrapolations from laboratory data and estimates of real-world variability. In the primary analysis, an assumption of an average inactivation of 3 log10 (99.9%) with a standard deviation of 0.6 log10 was used. However, actual home cooking conditions may have deviated from this assumption, especially when not thoroughly heated. Conservative scenarios (average inactivation of 2 log10) and strict scenarios (average inactivation of 4 log10) provided sensitivity boundaries. The uncertainty of this parameter mainly stemmed from the lack of inactivation data under large-scale real cooking conditions.

(4) Uncertainty of dose–response relationship.

The dose–response model used in this study was derived from experimental data from primates (Gao Shenyang et al.), and there were species differences when extrapolated to humans. At the same time, the model assumed that the susceptibility of the population was homogeneous, but in practice, the risk of infection in immunocompromised people, the elderly, and pregnant women may be significantly higher than that of healthy adults. We did not stratify these subgroups, which may have led to an underestimation of risk for high-risk groups.

(5) The uncertainty introduced by the variable independence assumption.

In the Monte Carlo simulation, we assumed that each input variable (e.g., prevalence, viral load, cooking cuts, and consumption) was independent the others. However, in reality, these variables may be correlated: for example, samples with high viral load tend to occur in a specific season, and shellfish consumption in that season may also rise in tandem with factors such as festivals; for example, cooking preferences may be geographically correlated, which in turn correlates with the level of contamination of primary shellfish. Ignoring these potential correlations can lead to biases in the estimation of the risk coalescence distribution. Due to the lack of sufficient joint data, we did not construct a correlation structure, which constituted the simplification assumption of this model.

## 4. Discussion

### 4.1. Key Findings and Their Public Health Implications

For the first time, based on the HEV pollution data of shellfish in China (2011–2024) and residents’ consumption patterns, a Monte Carlo was used to simulate the annual risk of HEV infection among urban and rural residents. The results showed that the median annual risk of HEV infection in urban and rural residents was 4.62 × 10^−6^ and 3.22 × 10^−6^, respectively, under the raw food scenario. This means that around 3–5 infections per million population per year may be associated with raw shellfish. Although this risk is low in absolute terms, the actual burden on certain groups of people may not be ignored given China’s large population base and the concentration of shellfish consumption in coastal areas.

Furthermore, cooking reduces the risk of infection by about three orders of magnitude (to the 10^−9^ level), which confirms the effectiveness of adequate heating as a key control point. This finding is consistent with the conclusions of the European Food Safety Authority (EFSA), which also emphasizes that cooking is the most effective means of blocking the transmission of foodborne HEV [16].

### 4.2. Comparison and Interpretation with Previous Studies

Previous studies on shellfish HEV have mostly been limited to contamination rate detection, and few studies have integrated pollution data with consumer behavior for quantitative risk assessment. Gao et al. [18] estimated the risk of infection based on early data close to the low quantile (2.5th percentile) of this study, but this study quantified the uncertainty range of risk for the first time and identified the key parameters driving risk through Bayesian updates and Monte Carlo simulations.

Sensitivity analysis showed that viral load and culinary inactivation were the most important factors affecting the final risk (Spearman ρ = 0.68 and −0.52). This means that: (1) source control (improving the breeding environment and reducing viral load) is a fundamental measure; (2) Endpoint control (ensuring adequate cooking) is the most effective means of protection that individuals can operate. In contrast, the effects of consumption frequency and single consumption are relatively small, suggesting that public health interventions should focus on changing cooking behavior rather than limiting consumption.

In terms of urban–rural differences, the cumulative annual risk of urban residents was slightly higher than that of rural residents due to higher frequency of consumption (λ = 104 times/year), while rural residents had a higher risk of single exposure due to greater single consumption (median 54.92 g). This finding suggests that risk communication strategies should be tailored to local conditions: urban areas can emphasize “sufficient heating for each consumption”, while rural areas need to remind residents to “control risk accumulation in a single large consumption”.

### 4.3. Methodological Innovation and Limitations

Innovations: The main methodological contributions of this study are: (1) integrating historical surveillance data and individual experimental data from 2011 to 2024, and improving the robustness of prevalence estimation through Bayesian updates; (2) the cooking inactivation effect was modeled probabilistically, and the ideal laboratory conditions and the variability of real home cooking were distinguished. (3) The complete R code and data (GitHub) are made public, making the research fully reproducible.

Despite efforts to address multiple uncertainties, this study has the following limitations: viral load data are of limited representativeness; the number of positive samples (n = 14) limits the tail accuracy of the fitted distribution, which is crucial for high quantile risk estimation. In the future, the scope of monitoring needs to be expanded, especially to cover different seasons and breeding areas.

A fixed conversion factor of 1:1000 was used in this study, but the actual ratio may fluctuate by 2–3 orders of magnitude. Due to the lack of reliable underlying data, we did not incorporate this uncertainty into the probabilistic model, which may lead to systematic bias in absolute risk values. However, this limitation does not affect the robustness of the core findings because the main conclusions of this study (e.g., protective effect of cooking, relative risk comparison between urban and rural areas) are not sensitive to the absolute value of the conversion ratio. More basic research on HEV infectivity detection is needed in the future to support more accurate translation models.

The index model recommended by EFSA is derived from primate experiments and is directly applied to species differences in humans. The model assumes that all populations have the same susceptibility, but in practice immunocompromised people, the elderly, and pregnant women may be at significantly higher risk. This study did not stratify these subpopulations and may have underestimated the burden in high-risk populations.

Monte Carlo simulations assume that input variables are independent of each other, but there may be correlations in reality (e.g., high pollution seasons overlapping with peak consumption seasons). Due to the lack of joint data, this study did not construct a relevant structure, which may lead to bias in the estimation of risk distribution.

The statistical yearbook does not disclose shellfish consumption data separately, and this study estimates exposure based on the total consumption of aquatic products and the proportion of literature reports, which introduces certain measurement errors. In the future, specialized shellfish consumption surveys will be needed to obtain more accurate exposure parameters.

### 4.4. Recommendations for Risk Management and Future Research

Based on the findings above, the following recommendations are proposed:

For regulatory authorities: (1) include HEV in the routine monitoring indicators for shellfish aquaculture waters and establish a pollution database; (2) explore using viral load as a potential grading indicator for shellfish safety to provide early warnings for high-risk batches; and (3) develop easy-to-understand shellfish cooking guidelines and promote the importance of “thorough heating” through multiple channels.

For consumers: especially residents in coastal areas, it is recommended to develop the habit of fully heating shellfish before consumption (e.g., boil until the shells open and continue heating for more than 5 min) and to avoid eating raw or lightly heated shellfish.

For researchers: (1) conduct large-scale surveys on shellfish consumption behavior to obtain accurate data on consumption frequency and cooking habits among urban and rural populations; (2) conduct studies on HEV inactivation kinetics under real cooking conditions to reduce uncertainty in cooking parameters; (3) explore the construction of multivariable risk models considering variable correlations; (4) conduct stratified risk assessments for high-risk subgroups; and (5) promote the standardization of HEV infectivity detection methods to provide data support for more accurate conversion rates.

The annual risk of HEV infection in Chinese residents from consuming shellfish was at the 10^−6^ level (raw) to 10^−9^ level (cooked), and thorough heating could reduce the risk by approximately 1000 times. Viral load and the inactivation effect of cooking were the main drivers of risk. The probabilistic risk assessment framework established in this study provided a scientific basis for shellfish food safety management, but more foundational data and refined models are needed to further reduce uncertainty.

## 5. Conclusions

This study integrated the contamination data of HEV in shellfish in China from 2011 to 2024 with the consumption data of residents, and for the first time used Monte Carlo simulation based on R language to quantitatively and probabilistically evaluate the infection risk of HEV among urban and rural residents exposed to shellfish through consumption in China.

The main conclusions are as follows:

Risk levels and cooking effectiveness: The median annual HEV infection risk under raw consumption scenarios was estimated at 10^−5^ (urban residents: 4.62 × 10^−6^; rural residents: 3.22 × 10^−6^). Adequate cooking (3 log_10_ virus reduction) reduced annual risk by approximately three orders of magnitude, demonstrating its critical role as a control measure.

Risk drivers: Sensitivity analysis identified viral load in shellfish and cooking effectiveness as the most influential parameters affecting infection risk. This highlights the importance of controlling viral contamination in aquaculture environments while promoting proper cooking practices among consumers.

Urban–rural differences: Urban residents exhibited higher cumulative annual risk due to greater consumption frequency, whereas rural residents faced higher per-exposure risk from larger serving sizes. These findings support the need for population-specific risk communication strategies.

Based on the above findings, we propose the following risk management recommendations:

For consumers, we should strengthen publicity and education, emphasizing that thoroughly heating and consuming shellfish is the most effective and controllable personal measure to prevent foodborne HEV infections.

For regulatory and industrial departments, it is recommended to strengthen the regular monitoring of HEV in water bodies and shellfish in major aquaculture production areas, and explore virus load as a potential key limiting indicator to reduce contamination levels from the source.

For future research, it is necessary to improve exposure parameters (such as precise consumption frequency and cooking habits) through larger-scale monitoring, and attempt to establish more realistic dose–response models to further enhance the accuracy of risk assessment.

In summary, this study established a quantitative framework for assessing HEV infection risk from shellfish and provided evidence-based recommendations for risk management strategies targeting both production and consumption stages of the food chain.

## Figures and Tables

**Table 1:** Annual infection risk for urban and rural residents under three different cooking scenarios (based on 100,000 Monte Carlo iterations).

	Scenarios	2.5th Percentile Risk (Risk Probability per Year)	Median Annual Risk (Risk Probability per Year)	97.5th Percentile Risk (Risk Probability per Year)	Scenario-Type
Urban Residents	Raw	3.18 × 10^−7^	4.63 × 10^−6^	6.88 × 10^−5^	conservative
	Cooked	1.02 × 10^−9^	4.65 × 10^−8^	2.03 × 10^−6^	conservative
	Raw	3.18 × 10^−7^	4.62 × 10^−6^	6.47 × 10^−5^	moderate
	Cooked	1.04 × 10^−10^	4.55 × 10^−9^	2.06 × 10^−7^	moderate
	Raw	3.24 × 10^−7^	4.63 × 10^−6^	6.78 × 10^−5^	stringent
	Cooked	1.01 × 10^−11^	4.64 × 10^−10^	2.18 × 10^−8^	stringent
Rural Residents	Raw	2.22 × 10^−7^	3.23 × 10^−6^	4.73 × 10^−5^	conservative
	Cooked	7.35 × 10^−10^	3.26 × 10^−8^	1.45 × 10^−6^	conservative
	Raw	2.29 × 10^−7^	3.22 × 10^−6^	4.56 × 10^−5^	moderate
	Cooked	7.33 × 10^−11^	3.19 × 10^−9^	1.45 × 10^−7^	moderate
	Raw	2.24 × 10^−7^	3.25 × 10^−6^	4.756 × 10^−5^	stringent
	Cooked	7.03 × 10^−12^	3.25 × 10^−10^	1.54 × 10^−8^	stringent

## Data Availability

The data that support the findings of this study are available on request from the corresponding author. All R code, simulation data, and results are available in a public GitHub repository: https://github.com/5484bg/Risk-Assessment.git, accessed on 28 January 2026.

## Abbreviations

The following abbreviations are used in this manuscript:

CI	Confidence interval
HEV	Hepatitis E virus
HE	Hepatitis E
WHO	World Health Organization
QMRA	Quantitative Microbial Risk Assessment
